# Potential of resistance inducers for citrus huanglongbing management via soil application and assessment of induction of pathogenesis-related protein genes

**DOI:** 10.1016/j.heliyon.2023.e19715

**Published:** 2023-09-01

**Authors:** Arya Widyawan, Mohammed A. Al-Saleh, Mahmoud H. El Komy, Hathal M. Al Dhafer, Yasser E. Ibrahim

**Affiliations:** Plant Protection Department, College of Food and Agriculture Sciences, King Saud University, Saudi Arabia

**Keywords:** Antibiotic, *Candidatus* liberibacter asiaticus, Gene expression, Salicylic acid, Soil drench, Phenylacetic acid

## Abstract

Huanglongbing (HLB) or citrus greening currently is the most devastating citrus disease worldwide. Unfortunately, no practical cure has been available up to now. This makes the control of HLB as early as possible very important to be conducted. The objective of this study was to investigate the efficacy of the application of salicylic acid (SA) and Phenylacetic acid (PAA) on one-year-old seedlings of different citrus species (*Citrus reticulata*, *C. sinensis*, *C. aurantifolii*) growing on *C. volkameriana* and *C. aurantium* by soil drench methods. Factorial analysis of variance showed the percent change in “*Candidatus* Liberibacter asiaticus” titer and disease severity on a different combination of citrus species growing on the two rootstocks treated with inducers and Oxytetracycline (OTC) were significantly different compared to the untreated plants. SA alone or in combination with OTC provided excellent (P-value < 0.05) control of HLB based on all parameters. The interaction between both factors (Rootstocks x Citrus species) significantly influenced the Ct value (P-value = 0.0001). “*Candidatus* Liberibacter asiaticus” titer in plants treated with OTC was reduced significantly with a range of −18.75 up to −78.42. Overall, the highest reduction was observed in the application of OTC on sweet orange growing on *C. volkameriana* (−78.42), while the lowest reduction was observed in the same cultivar which was treated with a combination of SA and OTC (−3.36). Induction of pathogenesis-related (*PR*) genes, i.e., *PR1*, *PR2*, and *PR15*, biosynthesis of Jasmonic acid and ethylene which are also important pathways to defense activity were also significantly increased in treated plants compared to untreated plants. This study suggests that the application of inducer alone is acceptable for HLB management. We proposed the application of SA and PAA as a soil drench on the citrus seedlings as promising, easy, and environmentally safe for HLB disease control on citrus seedlings.

## 1 Introduction

Citrus huanglongbing (HLB), also known as citrus greening, is one of the most destructive diseases of citrus in many citrus-producing countries [1]. The disease is widespread throughout Southern and Eastern Africa, in Asia from Pakistan to China [2,3], and also from South-to-North American countries [4,5]. Furthermore, the disease has also been observed in the southwestern part of the Arabian Peninsula [4]. It was probably introduced to Saudi Arabia in the early 1980s [4].

HLB disease is caused by an un-cultured and phloem-restricted bacterium known as *Candidatus* Liberibacter spp. Based on the *16S rRNA* DNA sequence, three species (‘*Candidatus* Liberibacter asiaticus' (CLas), ‘*Candidatus* Liberibacter africanus' (CLaf), and ‘*Candidatus* Liberibacter americanus' (CLam)) have been identified as causal agents of this disease [[6], [7], [8]]. HLB pathogens were transmitted from plant to plant with the help of an insect vector either *Diaphorina citri* (Kuwayama, 1908) or *Trioza erytreae* (Del Gurecio). The former is responsible for CLas transmission and has widespread throughout Asia, the Indian subcontinent, Saudi Arabia, Reunion, Mauritius, South and Central America, and Florida while the latter was responsible for CLaf transmission in Africa [9].

The HLB disease results in yellowing of the shoots, blotchy mottled leaves, corky veins, deformed and discolored fruit, premature fruit drop, root loss, and ultimately tree mortality [4,10,11]. Citrus trees that have been infected have a much shorter profitable life and production [10]. HLB reduces citrus tree production by up to 50%, and in advanced stages of infection, may cause the death of trees [12]. For example, since the disease was reported in Florida in 2005, it has spread to most citrus-producing regions, where it has resulted in the loss of about 100,000 acres of citrus and an estimated $3.6 billion in lost revenue [13]. According to current estimates, CLas has infected more than 95% of the mature trees in Florida's commercial citrus groves. Currently, there are no practical cures for HLB disease [1].

Different strategies and tactics have been implied in many citrus-producing countries with various results that have been shown to slow the progress of HLB and help maintain tree productivity under field conditions. These measures include the use of insecticides and biological control to control psyllid vectors [14,15], improved foliar nutritional programs [[16], [17], [18]], the application of inorganic phosphorus (P ) solution to the leaves [19], and the use of antibiotics like ampicillin, oxytetracycline, and streptomycin [[20], [21], [22]]. Additionally, soil conditioners [23], plant defense inducers or activators [21,24], different graft/rootstock combinations [25,26], and thermotherapy [27,28] may reduce the effects of HLB.

Inducers resistance, especially systemic acquired resistance application including salicylic acid (SA), oxalic acid, acibenzolar-*S*-methyl (ASM), and potassium phosphate via trunk injection, provides significant control of HLB disease by suppressing the pathogen titer and disease progress [21]. In addition, the application of SA and ASM via trunk injection showed significant induction in pathogenesis-related (*PR*) *PR-1* and *PR-2* genes [21].

Foliar applications of 2,6-dichloroisonicotinic acid (INA), β-aminobutyric, and benzothiadiazole, either individually or in combination, were successful in slowing the progression of HLB disease [24]. Previous studies found that the method of application of activators had a significant impact on their ability to suppress disease, with soil application being preferable to foliar spray [[29], [30], [31]]. Consequently, improving application treatments may lead to higher treatment outcomes. The purpose of this study was to evaluate SA and phenylacetic acid (PAA) applied individually by soil drench to young three citrus species; *C. sinensis* (Sweet orange), *C. reticulata* (Mandarin), and *C. aurantifolia* (Mexican lime) growing on *C. volkameriana* and *C. aurantium* on HLB disease progression under greenhouse conditions. Presently, no commercial citrus cultivar is resistant to the disease, and few effective measures are available for disease control. We believe that information on the control effect on suppressing the CLas population in plants, disease progress, and *PR* gene hopefully will provide valuable information on HLB management strategy, especially at the citrus young level (one-year-old).

## 2 Materials and methods

### 2.1 Plant material

One-year-old sweet orange, mandarin, and Mexican lime growing on *C. volkameriana* and *C. aurantium* rootstocks with an average trunk diameter of 9.53 mm and a budding height of 15.24–20.32 cm above the base of the stem grown under greenhouse conditions were grafted with symptomatic HLB buds (the presence of CLas was previously confirmed by PCR). In addition, different plants were grafted with pathogen-free buds, which were used as a control. Each cultivar was represented by two different rootstocks. Seedlings were grown in black plastic pots (20 cm diameter and 40 cm high) containing compost/perlite/vermiculite at 3:1:1 b y volume with a pH of six. After six months, these seedlings were then subjected to PCR confirmation of HLB presence (CLas). *Candidatus* Liberibacter asiaticus was detected using Quantitative PCR (qPCR) with a species-specific primer designed before [32] (Supplementary Table 1 and Table 1). Each sample was tested alone. Each sample corresponded to a single seedling and fully expanded symptomatic leaves (10 leaves) around the seedling was tested.

**Table 1:** Primers used for the detection of “*Candidatus* Liberibacter asiaticus” and Pathogenesis Related (*PR*), Jasmonic acid, and Ethylene biosynthesis gene expression analysis.

Objectives	Target	Encoding protein	Primer seq (5‵--3′)	References
*Candidatus* Liberibacter asiaticus	*rpo*B	β subunit-RNA polymerase	Forward: CGTCTCGTCAAGATTGCTATCCGT Reverse: TTAAGGACGCCCTTCTCTAGAAGG	[32]
PR gene analysis	*CsPR1*	Antifungal and antioomycetic	Forward: AACTCGCCTCAAGACTACCT Reverse: TGCAACTGTGTCGTTCCATA	[44]
	*CsPR2*	β 1,3 – glucanase	Forward: TTCCACTGCCATCGAAACTG Reverse: TGTAATCTTGTTTAAATGAGCCTCTTG	[29]
	*CsPR15*	Oxalate oxidase	Forward: TGGTTTGAGCAAAGAGGGTAATA Reverse: AGAGTATTGAGGCCAGGAAATG	[21]
	*GADPH*	glyceraldehyde-3 phosphate dehydrogenase	Forward: GGAAGGTCAAGATCGGAATCAA Reverse: CGTCCCTCTGCAAGATGACTCT	[29]
Jasmonic acid (JA) biosynthesis	*OPR_3*	12-oxophytodienoate reductase 3	Forward ACAGGACCGATCAATACGGC Reverse: GAGGCCAAGTGCTTCAGGAT	[45]
	*Aos*	allene oxide synthase	Forward: CAACATGCCTCCTGGTCCTT Reverse: CGGGGTTCTTACCGAACCAA	[45]
	*Lox*	Lipoxygenases	Forward: CCACGGTGTGACACAGATCA Reverse AGGATCGACAAAGGCGGTTT	[45]
Ethylene biosynthesis	*Ein*	ethylene insensitive-2	Forward: GAAAAGGATGATGATGAAGCAGATT Reverse: GAAGCCGGACCATCAGACAT	[46]
	*Acs*	1-aminocyclopropane-1-carboxylate synthase-1	Forward: TTCGAATCCACTAGGCACAACTT Reverse: CAACGCTCGTGAACTTAGGAGA	[46]
	*Etr*	ethylene response-1	Forward: TCGTCAGCAGAATCCTGTTGG Reverse: GGCCTTAATCTTGCTACTGGACA	[46]

### 2.2 Treatment applications

The chemical inducers applied in this experiment were SA [The SEARLE Company Ltd, Pakistan] and PAA [Sigma-Aldrich, St. Louis, MO, USA]. The inducer was applied as soil drench with the rate of 3 mg/ml and the volume applied was 100 ml per plant. The Antibiotic used in this experiment was Oxytetracycline (OTC) [Fisher Scientific- Pittsburgh, PA, USA] with a rate of 5 mg/ml, and the volume applied was 100 ml per plant. Inducers and antibiotics were applied as individuals or in combination with four times applications for each trial with an interval between applications of 15 days. The first treatment was applied two weeks after acclimatization.

Treatments were arranged in a randomized complete block design (RCBD) with six treatments including SA, SA + OTC, PAA, PAA + OTC, OTC, and infected-untreated control (IUTC), replicated three times in blocks of two contiguous seedlings (6 seedlings × 6 treatments × 3 cultivars × 2 rootstocks). The infected untreated control seedlings received a water-only. The plants were then maintained in a screened greenhouse at temperatures of 25–30 °C, 60 ± 5% relative humidity, and L16:D8 h photoperiod. Routine fertilization and irrigation schedules were applied after the treatments.

### 2.3 Disease assessment

With modification, a visual assessment of disease severity was conducted using the HLB disease severity scale described by Gottwald et al., [33]. The disease severity was assessed after four months of the first treatment based on a scale from 0 to 5, where 0 = none, 1 = ≤ 20, 2 = 21 up to 40, 3 = 41 up to 60, 4 = 61 up to 80, and 5 = 81 up to 100% of leaves affected. The means of the scale was calculated as the average of disease severity. Disease severity was expressed as a standardized area under the disease progress curve (AUDPC) using Simko and Piepho's formula [34].

### 2.4 DNA isolation and pathogen quantification

Due to the uneven distribution of the pathogen in the plant tissues, composite samples for pathogen quantification were performed. The sample was obtained from 3 to 5 leaves that showed HLB-like symptoms. For DNA extraction, a hundred milligrams of samples of leaf tissue midrib were minced and used for pathogen quantification (Doyle and Doyle [35]). Briefly, the midribs were frozen in liquid nitrogen and quickly ground to a fine powder with a mortar and pestle. The powder was incubated at 65 °C for 30 min in 1.5-ml microfuge tubes containing 1 ml of extraction buffer (100 mM TrisHCl, 50 mM EDTA, and 1.4 M NaCl), 100 μl of 10% cetyl trimethyl ammonium bromide (CTAB) and 75 μl of 10% sodium dodecyl sulfate (SDS). Samples were centrifuged at 14,000 rpm for 10 min. The supernatant was transferred to new microfuge tubes, where 200 μl of 3 M sodium acetate was added. The tubes were incubated on ice for 10 min and then centrifuged at 14,000 rpm for 10 min at room temperature. The clear supernatant was collected in a new microfuge tube and the aqueous phase was extracted using an equal volume of chloroform-isoamyl alcohol (24:1). The DNA was precipitated with isopropyl alcohol, washed with 70% ethyl alcohol, and then resuspended in 50 μl of DNAse free water. DNA was stored at −80 °C until used.

An aliquot of 3 μl (100 ng/μl) was used as a DNA template to determine pathogen titer using qPCR performed in two replicates on ABI 7500 [Applied Biosystem]. PCR mixtures were performed with a total volume of 20 μl; containing 10 μl of SYBR green PCR master mix, 250 nM of each primer, and template DNA. The real-time PCR was conducted at 95 °C initial denaturation for 5 min, followed by 40 cycles of 94 °C for 60 s and 60 °C for 30 s, with fluorescence signal capture at the end of each 62 °C step followed by a default melt (dissociation) stage. CLas-infected leaf samples of one isolate from Florida greenhouse-maintained CLas-citrus cultures were included as positive CLas controls (Provided by Dr. Judith K. Brown, the School of Plant Sciences (plant viruses and insect molecular diagnostic lab services), The University of Arizona). The effect of treatments was determined by comparing the percent changes in pathogen titer following the formula [(mean Cq value of pathogen after treatments - mean Cq value pathogen prior treatment)/mean Cq value pathogen prior treatment] x 100 as mentioned before [21].

### 2.5 Defense enzymes analysis and reactive oxygen species (ROS)

#### 2.5.1 Enzyme extraction

Leaves were cut from the citrus plants, washed with running tap water and weighted. Leaves (0.5 g) were immersed in liquid nitrogen and ground using mortar and pestle. The powder was resuspended in 1.2 ml Phosphate buffer (100 mM, pH 6.0) and incubated overnight. The homogenate was centrifuged at 12,000 rpm for 20 min. The supernatant was collected and used for further analysis [36,37].

#### 2.5.2 Peroxidase assay

Peroxidase (POX) activity was assayed according to Ramanathan et al. [39], with modification at room temperature. The reaction mixture contains 50 mM pyrogallol in 50 mM phosphate buffer (pH 6), enzyme extract and 3% of hydrogen peroxide as initiator. Directly after adding the initiator, enzyme activity was measured as change of absorbance at wave length 430 nm for 1 min [40]. Peroxidase activity was expressed as change of absorbance (Δ A_430_/min/gram fresh leaves).

#### 2.5.3 Polyphenol oxidase assay

Polyphenol oxidase (PPO) activity was assayed at room temperature. Catechol was used as a substrate for the enzyme [41]. The reaction mixture consists of 50 mM catechol in 50 mM phosphate buffer (pH 6.5) and enzyme extract. The mixture was incubated at 38 °C for 1 hour [42]. Polyphenol oxidase activity units per gram leaf was expressed as change of absorbance at 490 nm (Δ A_490_/min/gram fresh leaves) [41].

#### 2.5.4 Hydrogen peroxide (H_2_O_2_) assay

Leaf samples (0.5 g) were homogenized in 0.1% Tricholoaceric acid (TCA) and centrifuge at 12,000 rpm for 5 min at 4 °C. Supernatant (0.3 ml) was mixed with 1.7 ml potassium phosphate buffer (pH 7.0) and 1 ml of 1 M potassium iodide (KI) solution and incubated for 5 min. Post incubation, oxidation product was measured at A_390._ Hydrogen peroxide concentration was calculated from standard curve prepared before from known concentration of H_2_O_2_ and expressed as μmol/g [38].

### 2.6 Pathogenesis-related (*PR*) genes expression

Pathogenesis-related genes analyses were conducted at four different times following the treatments which were 0, 1, 3, and 5 days after treatments on two citrus cultivars only sweet orange (highly susceptible) and Mexican lime (moderate resistant). At a given sampling point, the composite sample obtained from 3 to 5 leaves showing HLB symptoms was collected. The leaves were immediately frozen in liquid nitrogen, powdered, and kept at −80 °C until further analysis. Total RNA was extracted using Triazole ™ Reagent following the instruction provided by the company. Briefly, 100 mg of leaves tissue powder was mixed with 1 ml Triazole ™ Reagent and homogenated following the incubation at room temperature for 5 min 0.2 ml of chloroform was added to the mixture and incubated for 2–3 min. The mixture was centrifuged at 12,000×*g* at 4 °C for 15 min to separate it into lower red phenol-chloroform, interphase, and a colorless upper aqueous phase that contains the RNA. The RNA was precipitated with isopropanol and washed with 75% ethanol. RNA was resuspended in nuclease-free water and stored at −80 °C until further analysis. An aliquot of 3 μl total RNA (25 ng/μl) of total RNA was used for cDNA synthesis. cDNA was synthesized using the OneScript® Plus cDNA Synthesis Kit [abm, Canada]. Following, 1.5 μl of cDNA was mixed with SYBR green PCR master mix for gene expression of *PR1*, *PR2*, *PR15*, Jasmonic acid (JA) biosynthesis gene, and Ethylene biosynthesis quantified using ABI 7500 [Applied Biosystem]. The housekeeping gene encoding glyceraldehyde-3 phosphate dehydrogenase-C (*GADPH-C*) was used as an endogenous control. Primers used in this study are summarized in Table 1. Relative gene expression was calculated using the 2^−ΔΔCq^ method [43]. Briefly, the fold change in the target gene was normalized to plant *GADPH-C* gene and relative to the expression at time 0 was calculated for each sample using the equation ΔΔCq = (Cq target – Cq GADPH) Time x - (Cq target – Cq GADPH) Time 0.

### 2.7 Statistical analysis

Prior to variance analysis, the data were first tested for normality of variance using Levene's test. Data collected from two trials were pooled when no significant differences were found according to Bartlett's test (P-value > 0.05). To determine the effect of treatments, cultivars, and rootstock on disease severity, pathogen titer, and PR gene expression, the means were analyzed using factorial ANOVA; treatments vs. cultivars, treatments vs. rootstock, and cultivars vs. rootstock vs. treatments. Following the ANOVA analysis, the least significant difference test was conducted to separate the means. The test was conducted using Fisher LSD at P-value < 0.05. Statistical analysis was conducted using Statistix 8.1 [Statistix 8, 2003; Analytical Software, Tallahassee, FL].

## 3 Results

### 3.1 Detection of “*Candidatus* Liberibacter asiaticus” prior to treatments

One week prior to the start of the trial, CLas titers using qPCR assay were determined. The Cq values of the CLas were varying between 19.33 and 35.23 (Supplementary Table 1). The former was detected on sweet orange/*C. volkameriana* and the latter were detected on Mexican lime/*C. aurantium.* Factorial ANOVA analysis showed that citrus species and rootstock as a single and the interaction between both factors (Rootstocks x Citrus species) significantly influenced the Ct value (P-value <0.05) (Table 2).

**Table 2:** Factorial analysis of variance of “*Candidatus* Liberibacter asiaticus” (CLas) -titer reflected as Ct value prior to treatments and percent (%) change in CLas population after treatments.^a^

Source of variance	SS	Df	MS	F	P-value^a^
**Prior to treatments**					
Rootstocks	67,6256	1	67,6256	3,91	**0.0003451**
*Citrus* species	248.287	2	124.1435	2,68	**3.205e-10**
Rootstocks x *Citrus* species	609.618	2	304.809	9,67	**0,00001**
Error	1072.6956	210			
Total	1998.2262	215			
**Post to treatments**					
*Citrus species*	792.4109	2	396.2054	2.78	**0.06446**
Treatments	31203.7569	5	6240.7514	43.7884	**0,00001**
*Citrus* species x Treatments	8354.9352	10	835.4935	5.8623	**9.791e-8**
Error	28219.0803	198	142.5206		
Total	68570.1832	215			
Rootstocks	158.0382	1	158.0382	0.8969	0.3447
Treatments	31203.7569	5	6240.7514	35.4193	**0,00001**
Rootstock x treatments	1264.3172	5	252.8634	1.4351	0.213
Error	35944.0709	204	176.1964		
Total	68570.1833	215			
Rootstock x *Citrus* species	6541.9782	5	1308.3956	28.5378	**0,00001**
Treatments	31203.7569	5	6240.7514	136.1189	**0,00001**
Treatments x Rootstock x *Citrus* species	22571.848	25	902.8739	19.6929	**0,00001**
Error	8252.6002	180	45.8478		
Total	68570.1832	215			

a P-value in bold showed a significant effect of source variant against Clas titer and % change in CLas population (P-value < 0.05)

### 3.2 Effects of treatments on “*Candidatus* Liberibacter asiaticus” titer and disease severity

Generally, the application of inducer alone or in combination with antibiotic significantly reduced pathogen titer (Table 3) and disease severity expressed as Area Under Disease Progress (Table 4) compared to untreated plants (treated with water only). Percent changes of pathogen titer in plants treated with inducer alone and in combination with antibiotic were varies between −3.36% and −30.34%. A negative value on this parameter indicated a reduction in the pathogen titer. On the other hand, CLas titer in plants treated with water only increased with percent changes between 4.25 and 7.20. Meanwhile, CLas titer in plants treated with antibiotic was reduced significantly with a range of −18.75 up to −78.42. Overall, the highest reduction was observed in the application of antibiotic on sweet orange growing on *C. volkameriana* (−78.42), while the lowest reduction was observed in the same cultivar which was treated with a combination of SA and antibiotic (−3.36). (Supplementary Table 2).

**Table 3:** The percent changes in “*Candidatus* Liberibacter asiaticus” titers in leaf samples (mean Cq value of CLas after treatments - mean Cq value of CLas prior treatment)/mean Cq value CLas prior treatment] x 100) of Sweet orange, Mandarin, and Mexican lime growing on *Citrus volkameriana* and *C. aurantium* seedlings treated with salicylic acid (SA) and phenylacetic acid (PAA) in a one-year seedling under greenhouse conditions.

Cultivar/rootstock	without OTC^a^		with OTC		Controls		Critical Value
	SA	PAA	SA	PAA	OTC	IUTC^b^	
Mandarin/*C. volkameriana*	−12.25 ± 1.6 bce	−10.32 ± 1.5 c	−6.65 ± 0.53 c	−18.2 ± 5.48 ab	−20.23 ± 4.70 a	6.99 ± 0.3 d	7.6
Sweet orange/*C. volkameriana*	−23.34 ± 7.2 b	−18.62 ± 3.8 b	−3.36 ± 0.45 bce	−9.25 ± 3.70 bce	−78.42 ± 18.8 a	4.5 ± 1.84 c	20.82
Mexican lime/*C. volkameriana*	−21.03 ± 2.4 a	−22.05 ± 0.6 a	−24.96 ± 0.65 a	−23.01 ± 2.37 a	−24.23 ± 6.40 a	5.04 ± 1.7 b	7.48
Mandarin/*C. aurantium*	−25.33 ± 5.1 bce	−18.31 ± 5.1 c	−17.04 ± 3.7 c	−30.34 ± 3.04 b	−47.85 ± 3.97 a	5.55 ± 2.0 d	9.7
Sweet orange/*C. aurantium*	−19.93 ± 3.5 a	−19.81 ± 6.6 a	−12.37 ± 1.33 a	−14.51 ± 4.36 a	−21.14 ± 4.44 a	7.20 ± 1.4 b	9.9
Mexican lime/*C. aurantium*	−8.79 ± 1.23 b	−7.77 ± 2.74 b	−7.53 ± 1.07 b	−9.83 ± 2.01 b	−18.75 ± 3.36 a	6.77 ± 2.0 c	5.46

*) value with same letter in row showed no significant diference.; Means separation was conducted using Fisher LSD α = 0.05; Negative value indicating reduction *Candidatus* Liberibacter asiaticus titers.

a OTC = oxytetracycline.

b IUTC = infected untreated control.

**Table 4:** Citrus huanglongbing disease severity, expressed as a standardized area under disease progress curve, achieved by salicylic acid (SA), and phenylacetic acid (PAA) treatments in a one-year seedling under greenhouse conditions.

Cultivar/rootstock	without OTC^a^		with OTC		Controls		Critical Value
	SA	PAA	SA	PAA	OTC	IUTC^b^	
Mandarin/*C. volkameriana*	0.415 ± 0.02 c	0.41 ± 0.02 c	0.98 ± 0.07 b	0.25 ± 0.03 d	0.415 ± 0.02 c	1.8 ± 0.23 a	0.85
Sweet orange/*C. volkameriana*	0.915 ± 0.02 bce	1.18 ± 0.4 ab	0.33 ± 0.07 e	0.68 ± 0.07 cd	0.44 ± 0.014 de	1.3 ± 0.2 a	0.56
Mexican lime/*C. volkameriana*	0.58 ± 0.07 b	0.44 ± 0.01 b	0.48 ± 0.07 b	0.44 ± 0.014 b	0.48 ± 0.07 b	0,8 ± 0.21 a	0.42
Mandarin/*C. aurantium*	0.68 ± 0.35 b	0.68 ± 0.35 b	0.35 ± 0.11 b	0.32 ± 0.16 b	0.31 ± 0.17 b	1.7 ± 0.2 a	0.61
Sweet orange/*C. aurantium*	0.25 ± 0.023 c	0.25 ± 0.04 c	1.02 ± 0.12 b	0.45 ± 0.14 c	0.245 ± 0.02 c	1.3 ± 0.21 a	0.86
Mexican lime/*C. aurantium*	0.76 ± 0.04 a	0.44 ± 0.01 b	0.42 ± 0.02 b	0.26 ± 0.04 c	0.25 ± 0.028 c	0.8 ± 0.21 a	0.32

*) value with the same letter in a row showed no significant difference. Means separation was conducted using Fisher LSD α = 0.05.

a OTC = oxytetracycline.

b IUTC = infected untreated control.

Coherence with analysis of variance prior to treatments, and analysis of variance after treatments showed that Citrus species and rootstocks as a single factor did not significantly affect the percent change in the CLas titer with P-value 0.47 and 0.39 respectively. Meanwhile, the interaction between both factors (rootstock x Citrus species) significantly affects the percent change in CLas titer after treatments (P-value 0.0000) (Table 2). All applications of inducer alone and in combination with antibiotic showed promising results since there was no significant difference with the application of antibiotic alone with sweet orange growing on *C. aurantium* and Mexican lime growing on *C. volkameriana*, while in mandarin growing on *C. volkameriana*, only application of PAA in combination with antibiotic showed no significant with the application of antibiotic alone. In the case of mandarin and Mexican lime growing on *C. volkameriana* and *C. aurantium*, the application of PAA in combination with antibiotic gave a percent reduction at −30.43 and −9.83 which were the highest among other applications, although the reduction was not significant compared to the application of antibiotic alone; −47.85 and −18.75 for mandarin and Mexican lime growing on *C. volkameriana* respectively. Application of inducer alone (SA and PAA) and SA in combination with antibiotic showed promising results on Mexican lime and sweet orange growing on *C. volkameriana*. The highest percent (−25.33 and −22.05) reduction was re-ported in the case of the application of SA, PAA, and SA in combination with antibiotic on mandarin and Mexican lime growing on *C. volkameriana; re*spectively.

The application of inducers alone and in combination with antibiotic reduced the severity of the treated plant compared to untreated plants (Table 4). The severity of the treated plant with the inducer alone and in combination with antibiotic was 0.25 up to 1.02. This value was smaller than the severity of plants treated with water with a range of 0.8 up to 1.8. Meanwhile, the severity of plants treated with antibiotic was in the range of 0.245 up to 0.48. Compared to plants treated with water only, all treatments with inducer alone or in combination with antibiotic showed a significant effect on Mandarin with rootstock *C. aurantium* with values 0.35 up to 0.68 and Mexican lime with rootstock *C. volkameriana* with values 0.44 up to 0.58. Meanwhile, in others, the significance was variable. Application of inducer (SA or PAA) alone showed significant results on Mandarin/*C. volkameriana* and sweet orange/*C. aurantium* with values of 0.415 and 0.25 respectively. Application of SA and PAA in combination with antibiotic showed significance in all treated plants with a range value from 0.25 up to 1.02.

### 3.3 ROS and defense enzymes

In general, ROS activity and defense enzymes including H_2_O_2_, peroxidase and polyphenol oxidase activity showed significant increasing. Hydrogen peroxide (H_2_O_2_) activity on treated infected plants showed significant increasing compared to untreated control (Table 5). The highest activity of H_2_O_2_ was 1.39 μmol/g, 1.58 μmol/g, 1.35 μmol/g, 1.1 μmol/g, and 1.51 μmol/g respectively for combination mandarin/*C. volkameriana*, mexican lime/*C. volkameriana*, mandarin/*C. aurantium*, sweet orange/*C. aurantium*, and mexican lime/*C. aurantium*. However, in combination of sweet orange/*C. volkameriana* no significant was found between treated plants and controls. Application of inducer alone gave highest H_2_O_2_ activity 1.35 μmol/g and 1.51 μmol/g respectively for SA and PAA. In combination with OTC, highest H_2_O_2_ activities were 1.39 μmol/g and 1.37 μmol/g respectively for SA and PAA.

**Table 5:** Hydrogen Peroxide (H_2_O_2_) activity (μmol/g) on infected citrus tree by Candidatus Liberibacter asiaticus and treated by salicylic acid (SA), and phenylacetic acid (PAA) treatments in a one-year seedling under greenhouse conditions.

Cultivar/rootstock	without OTC^a^		with OTC		Controls		Critical Value
	SA	PAA	SA	PAA	OTC	IUTC^b^	
Mandarin/*C. volkameriana*	1.15 ± 0.002 c	1.09 ± 0.005 d	1.39 ± 0.025 a	1.14 ± 0.004 c	1.32 ± 0.018 b	0.98 ± 0.006 e	0.053
Sweet orange/*C. volkameriana*	0.99 ± 0.012 a	1.23 ± 0.27 a	1.04 ± 0.055 a	0.97 ± 0.008 a	1.09 ± 0.098 a	0.89 ± 0.006 a	0.48
Mexican lime/*C. volkameriana*	1.52 ± 0.02 a	1.58 ± 0.07 a	1.11 ± 0.1 a	1.11 ± 0.052 a	1.64 ± 0.18 a	0,91 ± 0.024 b	0.36
Mandarin/*C. aurantium*	1.35 ± 0.016 a	0.88 ± 0.014 c	1.03 ± 0.026 b	0.83 ± 0.012 d	0.85 ± 0.01 cd	0.71 ± 0.012 e	0.063
Sweet orange/*C. aurantium*	0.844 ± 0.008 b	1.02 ± 0.07 a	1.07 ± 0.008 a	1.1 ± 0.06 a	1.04 ± 0.01 a	0.89 ± 0.007 b	0.15
Mexican lime/*C. aurantium*	1.33 ± 0.09 b	1.51 ± 0.012 a	1.11 ± 0.01 c	1.37 ± 0.002 b	1.34 ± 0.03 b	0.89 ± 0.001 d	0.16

*) value with the same letter in a row showed no significant difference. Means separation was conducted using Fisher LSD α = 0.05.

a OTC = oxytetracycline.

b IUTC = infected untreated control.

Polyphenol oxidase (PPO) activity on treated infected plants showed significant increasing compared to untreated control (Table 6). The highest activity of PPO was 0.5, 0.51, 0.51, 0.49, 0.62, and 0.51 activity/min/gram respectively for combination mandarin/*C. volkameriana*, sweet orange/*C. volkameriana*, mexican lime/*C. volkameriana*, mandarin/*C. aurantium*, sweet orange/*C. aurantium*, and mexican lime/*C. aurantium* respectively. Application of inducer alone gave highest PPO activity 0.62 and 0.5 activity/min/gram respectively for SA and PAA. In combination with oxytetracycline, highest PPO activities were 0.51 activity/min/gram for SA and PAA.

**Table 6:** Polyphenol oxidase (PPO) activity on infected citrus tree by Candidatus Liberibacter asiaticus and treated by salicylic acid (SA), and phenylacetic acid (PAA) treatments in a one-year seedling under greenhouse conditions.

Cultivar/rootstock	without OTC^a^		with OTC		Controls		Critical value
	SA	PAA	SA	PAA	OTC	IUTC^b^	
Mandarin/*C. volkameriana*	0.47 ± 0.003 a	0.49 ± 0.003 a	0.5 ± 0.004 a	0.48 ± 0.001 a	0.482 ± 0.01 a	0.37 ± 0.025 b	0.044
Sweet orange/*C. volkameriana*	0.51 ± 0.014 a	0.47 ± 0.017 a	0.47 ± 0.038 a	0.48 ± 0.04 a	0.51 ± 0.037 a	0.37 ± 0.024 b	0.12
Mexican lime/*C. volkameriana*	0.51 ± 0.06 a	0.5 ± 0.06 a	0.51 ± 0.056 a	0.5 ± 0.056 a	0.51 ± 0.058 a	0.33 ± 0.03 b	0.216
Mandarin/*C. aurantium*	0.45 ± 0.03 a	0.47 ± 0.01 a	0.49 ± 0.01 a	0.49 ± 0.0012 a	0.48 ± 0.01 a	0.37 ± 0.03 b	0.072
Sweet orange/*C. aurantium*	0.62 ± 0.026 a	0.47 ± 0.017 b	0.46 ± 0.02 b	0.5 ± 0.015 b	0.47 ± 0.02 b	0.37 ± 0.024 c	0.078
Mexican lime/*C. aurantium*	0.48 ± 0.021 b	0.5 ± 0.04 a	0.48 ± 0.001 b	0.51 ± 0.03 a	0.51 ± 0.01 b	0.32 ± 0.035 b	0.132

*) value with the same letter in a row showed no significant difference. Means separation was conducted using Fisher LSD α = 0.05.

a OTC = oxytetracycline.

b IUTC = infected untreated control.

Peroxidase (POX) activity on treated infected plants showed significant increasing compared to untreated control (Table 7). The highest activity of POX was 0.52, 0.53, 0.52, 0.52, 0.52, and 0.51 activity/min/gram respectively for combination mandarin/*C. volkameriana*, sweet orange/*C. volkameriana*, mexican lime/*C. volkameriana*, mandarin/*C. aurantium*, sweet orange/*C. aurantium*, and mexican lime/*C. aurantium*. Application of inducer alone gave highest POX activity 0.62 and 0.5 activity/min/gram respectively for SA and PAA. In combination with OTC, highest POX activities were 0.51 activity/min/gram for SA and PAA.

**Table 7:** Peroxidase (POX) activity on infected citrus tree by Candidatus Liberibacter asiaticus and treated by salicylic acid (SA), and phenylacetic acid (PAA) treatments in a one-year seedling under greenhouse conditions.

Cultivar/rootstock	without OTC^a^		with OTC		Controls		Critical value
	SA	PAA	SA	PAA	OTC	IUTC^b^	
Mandarin/*C. volkameriana*	0.49 ± 0.002 a	0.5 ± 0.005 a	0.52 ± 0.016 a	0.5 ± 0.005 a	0.5 ± 0.003 a	0.34 ± 0.02 b	0.044
Sweet orange/*C. volkameriana*	0.5 ± 0.006 a	0.48 ± 0.014 a	0.5 ± 0.02 a	0.5 ± 0.007 a	0.53 ± 0.006 a	0.34 ± 0.03 b	0.0695
Mexican lime/*C. volkameriana*	0.49 ± 0.012 a	0.49 ± 0.016 a	0.5 ± 0.006 a	0.49 ± 0.003 a	0.49 ± 0.01 a	0.35 ± 0.005 b	0.0402
Mandarin/*C. aurantium*	0.52 ± 0.013 a	0.5 ± 0.03 a	0.49 ± 0.003 a	0.5 ± 0.006 a	0.51 ± 0.003 a	0.36 ± 0.013 b	0.0321
Sweet orange/*C. aurantium*	0.48 ± 0.026 a	0.51 ± 0.017 a	0.49 ± 0.022 a	0.52 ± 0.004 a	0.5 ± 0.021 a	0.33 ± 0.02 b	0.0742
Mexican lime/*C. aurantium*	0.5 ± 0.007 a	0.51 ± 0.02 a	0.5 ± 0.003 a	0.51 ± 0.03 a	0.49 ± 0.004 a	0.34 ± 0.024 b	0.0529

*) value with the same letter in a row showed no significant difference. Means separation was conducted using Fisher LSD α = 0.05.

a OTC = oxytetracycline.

b IUTC = infected untreated control.

### 3.4 Pathogenesis related (PR) gene expression

**Fig. 1:**
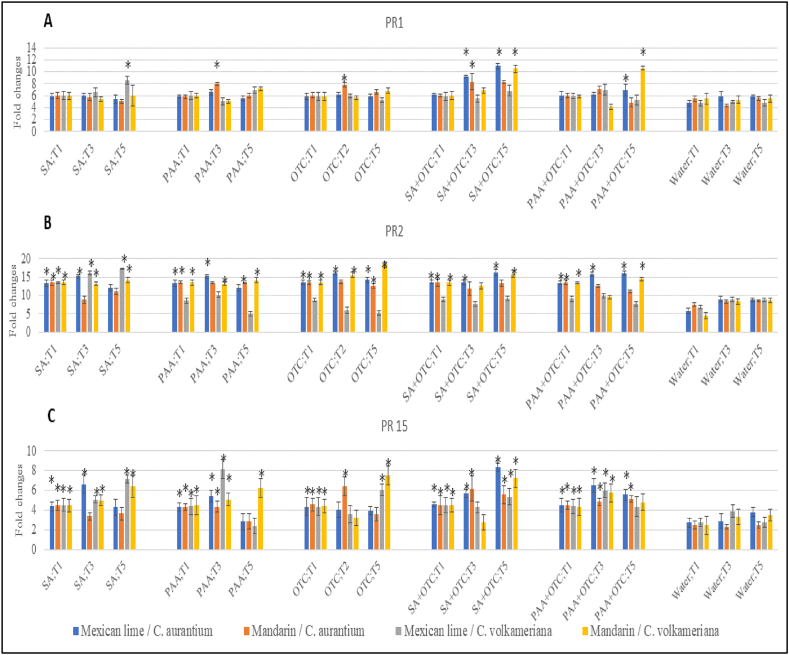
Relative expression of pathogenesis-related (*PR*) genes *PR-1* (panel A), *PR-2* (panel B), and *PR-15* (panel C) in young seedlings of mexican lime/*C. aurantium*, mandarin/*C. aurantium*, mexican lime/*C. volkameriana*, and mandarin/*C. volkameriana* after treated with SA and PAA. The relative gene expression was calculated as fold change using the 2^−ΔΔCq^ method. Values are means of three biological replicates and each sample consisted of five pooled leaves from a treated tree. Error bars represent standard error. Asterisks indicate a significant difference (P-value < 0.05, Fisher LSD) between treatments and water treated only. Abbreviations: SA = salicylic acid, PAA = Phenylacetic acid; OTC = Oxytetracycline; T1 = 1 day after treatments, T3 = 3 days after treatments, T5 = 5 days after treatments.

Besides ROS and defense enzyme effect, effect of SA or PAA alone and in combination with OTC also evaluated on expression of defense genes such *PR* genes, biosynthesis of Jasmonic Acid (JA) and Ethylene. Application of SA or PAA alone and in combination with OTC increased the expression of *PR1*, *PR2*, and *PR 15* significantly compared to untreated plants (Fig. 1). Application of SA significantly induced the expression of *PR1* in the range of 8.58 fold changes up to 10.53 fold changes; *PR2* in range of 12.07 up to 16.2 fold changes; and *PR15* in range of 4.28 up to 8.34 fold changes. Meanwhile, PAA induced significant induction of the expression of *PR1* in 7.94 fold changes up to 10.60 fold changes; *PR2* in range of 13.27 up to 16.08 fold changes; and *PR15* in range of 4.33 up to 6.5 fold changes.

**Fig. 2:**
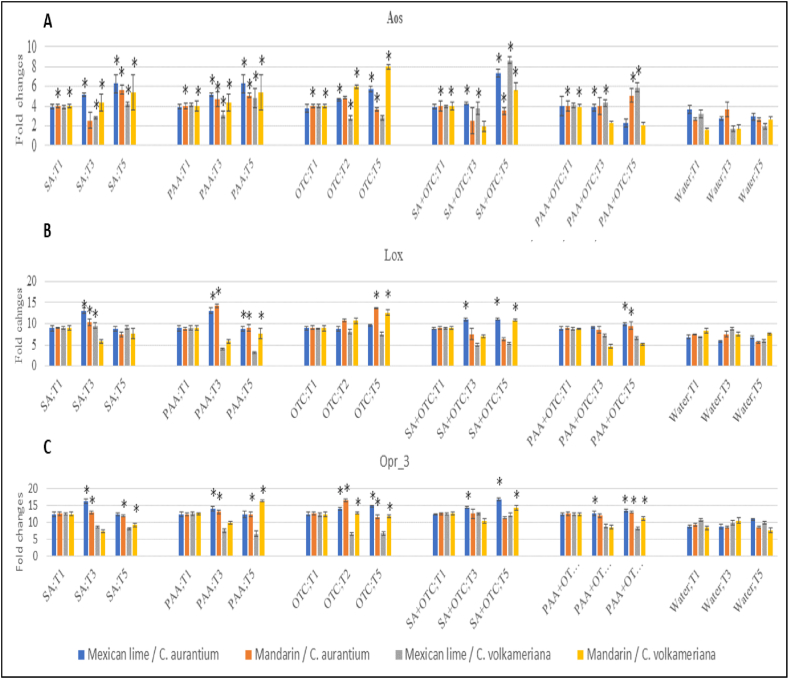
Relative expression of Jasmonic acid (JA) biosynthesis genes *Aos* (panel A), *Lox* (panel B), and *Opr_3* (panel C) in young seedlings of mexican lime/*C. aurantium*, mandarin/*C. aurantium*, mexican lime/*C. volkameriana*, and mandarin/*C. volkameriana* after treated with SA and PAA. The relative gene expression was calculated as fold change using the 2^−ΔΔCq^ method. Values are means of three biological replicates and each sample consisted of five pooled leaves from a treated tree. Error bars represent standard error. Asterisks indicate a significant difference (P-value < 0.05, Fisher LSD) between treatments and water treated only. Abbreviations: SA = salicylic acid, PAA = Phenylacetic acid; OTC = Oxytetracycline; T1 = 1 day after treatments, T3 = 3 days after treatments, T5 = 5 days after treatments.

Applications of SA or PAA alone and in combination with OTC also increased the expression Jasmonic Acid (JA) biosynthesis genes including *Aos*, *Lox*, and *Opr_3* significantly compared to untreated plants (Fig. 2). Application of SA significantly induced the expression of *Aos* in range of 2.84 up to 8.63 fold changes; *Lox* in range of 9.49 up to 13.03 fold changes; and *Opr_3* in range of 9.2 up to 16.88 fold changes. Meanwhile, PAA induced significant induction of the expression of *Aos* in range of 3.14 up to 6.25 fold changes; *Lox* in range of 8.87 up to 14.26 fold changes; and *Opr_3* in range of 11.15 up to 16.534 fold changes.

**Fig. 3:**
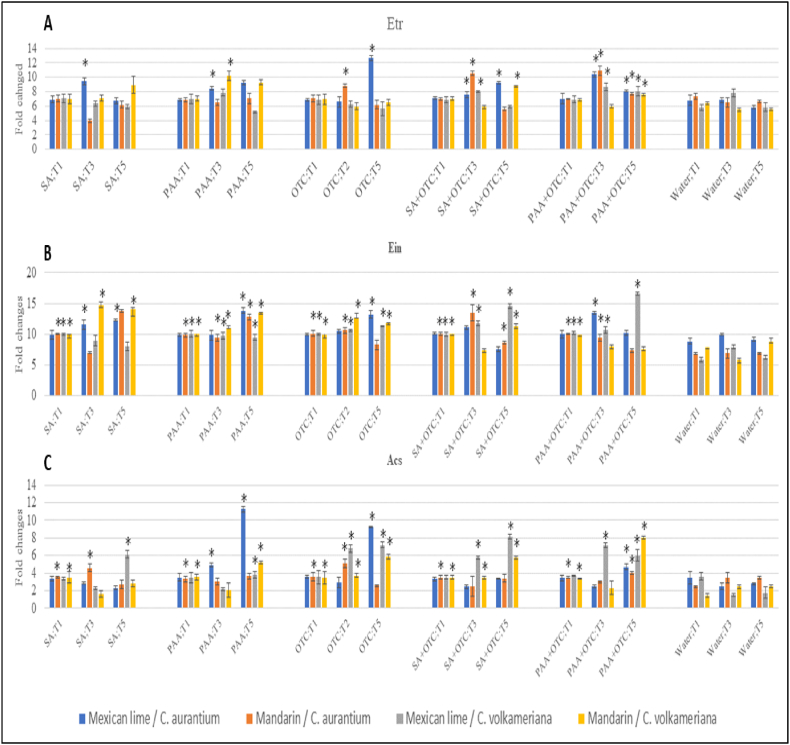
Relative expression of Ethylene biosynthesis genes *Etr* (panel A)*, Ein* (panel B), and *Acs* (panel C), in young seedlings of mexican lime/*C. aurantium*, mandarin/*C. aurantium*, mexican lime/*C. volkameriana*, and mandarin/*C. volkameriana* after treated with SA and PAA. The relative gene expression was calculated as fold change using the 2^−ΔΔCq^ method. Values are means of three biological replicates and each sample consisted of five pooled leaves from a treated tree. Error bars represent standard error. Asterisks indicate a significant difference (P-value < 0.05, Fisher LSD) between treatments and water treated only. Abbreviations: SA = salicylic acid, PAA = Phenylacetic acid; OTC = Oxytetracycline; T1 = 1 day after treatments, T3 = 3 days after treatments, T5 = 5 days after treatments.

Application of SA or PAA alone and in combination with OTC also increased the expression of Ethylene biosynthesis genes; *Etr, Ein*, and *Acs* significantly compared to untreated plants (Fig. 3). Application of SA significantly induced the expression of *Etr* in the range of 8.01 fold changes up to 10.54-fold changes; *Ein* in the range of 9.85 fold changes up to 14.66 fold changes; and *Acs* in range of 3.48 up to 8.12 fold changes. Meanwhile, PAA induced significant induction of the expression of *Etr* in 8.06 fold changes up to 10.88 fold changes*; Ein* in 9.41 fold changes up to 16.59 fold changes; and *Acs* in range of 3.34 up to 11.26 fold changes.

## 4 Discussion

The present study evaluated the application of SA and PAA alone and in combination with OTC on three commercial cultivars, i.e., sweet orange, mandarin, and Mexican lime growing on *C. volkameriana* and *C. aurantium* under greenhouse conditions. The inducers were delivered inside the plants through a soil drench application. Although previous studies indicated that the soil drench application showed poor absorption and translocation of the inducer, others suggested that the application of the inducer as a soil drench provides effective protection against bacterial disease [29,31,47]. The induction of plant defense by chemical inducers is more effective for young trees with mild HLB symptoms than older trees with severe HLB symptoms [24]. Injection application showed excellent results for slowing down HLB disease progression [47], but this method is difficult for actual application because of the large cost of humans and materials [47].

Application of SA effectively reduced the disease progress of citrus canker [48], citrus green mold [49], and also citrus greening [21,47]. SA is an aromatic acid that is highly photodegradable and biodegradable. Moreover, SA may be vulnerable to rainfall wash-off and the chemical was also absorbed by soil particles so it reduces the uptake by roots [50,51]. However, we found that the application of SA on different citrus species with different rootstocks showed promising disease management results. Application of SA alone significantly reduced the CLas-titer compared to untreated plants. Moreover, in some combinations of citrus species and rootstocks such as Mexican lime/*C. volkameriana* and sweet orange/*C. aurantium*, the application of SA alone showed a significant reduction compared to the application of OTC alone, which means that SA is promising to be used as an alternative control to replace the antibiotic.

On the other hand, the application of PAA also showed a significant reduction in percent changes of CLas-titer compared to untreated plants. No significant reduction was reported in the case of SA alone with all combinations of citrus species and rootstock. Sáenz-Pérez et al. [52] reported that *C. aurantium* rootstock is not suitable for lemon and sweet orange since it had the highest level of CLas titer. However, *C. macropylla* and *C. volkameriana* showed lower Cq values. PAA is also known to be potential Induced Systemic Resistance (ISR) determinant that effectively protects against the Fusarium wilt disease of tomato [53,54] and the bacterial soft rot pathogen *Pectobacterium carotovorum* subsp. *Carotovorum* [55]. The application of PAA alone also significantly increases the PR gene's expression. Moreover, the application of PAA alone showed consistent significant expression of the PR 2 and PR15 genes at the combination of mandarin growing on *C. volkameriana* and *C. aurantium*. Our findings suggest that the application of PAA for the management of HLB is promising and suitable for almost all combinations of citrus species and rootstocks used in this experiment. To our knowledge, this is the first time to use PAA has been against HLB disease.

Oxytetracycline was widely used for bacterial diseases control since it was easy to apply through various methods such as soil drenching, foliar spray, or trunk injection [[56], [57], [58]], not produce phytotoxicity on citrus [59], and easily taken by the plant [60]. The minimum bactericidal concentration for OTC is more than four times the Minimal inhibitory concentration (MIC), and it is well known that it is bacteriostatic against a variety of gram-negative and gram-positive bacteria [61]. In this study, the combination of inducer and antibiotic was significantly effective in controlling the HLB pathogen and reducing disease severity compared to the untreated control. It may suggest that the combination of inducer and antibiotic is promising to be applied. However, more studies should be conducted to observe the efficacy and environmental safety of this combination because some studies in the pharmaceutical field suggest a drawback of the combination between SA and antibiotics. SA was known to be able to induce phenotypic resistance in bacteria by three different mechanisms including up or downregulating outer membrane proteins or efflux pumps, upregulating antibiotic targets, and inducing enzymes with degrading activity. SA can also increase the frequency of mutation resulting in antibiotic resistance [62].

Application of SA and PAA in HLB-infected citrus seedlings increased the expression of *PR1*, *PR2*, and *PR15* significantly with different combinations of citrus cultivars and rootstock. For example, the application of PAA increased the *PR2* and *PR15* genes expression significantly and consistently. Meanwhile, SA consistently increased the *PR2* and *PR15* gene expression and *PR1* in the combination with OTC. It is well known that the application of the inducer will increase the expression of the PR gene and trigger the defense mechanism which has two main pathways; the SA-dependent pathway and the Jasmonic acid (JA) dependent pathway. *PR1, PR2,* and *PR15* genes are examples of SA-dependent PR genes [[63], [64], [65]], while *PR3*, *PR4*, and *PR12* represent the JA-dependent pathway [66]. However, activators including oxalic acid, SA, potassium phosphate and ASM were not able to induce *PR-3* gene expression in the pathosystem of citrus and CLas [21]. *PR1*, *PR2*, and *PR15* have properties and function as antifungal, β-1,3-glucanases activity, and, oxalate oxidase [15]. Induction of *PR1* and *PR2* will trigger the defense mechanism against fungi and oomycetes, but the contribution of *PR1* and *PR2* in HLB disease remains unknown [21]. These earlier studies hypothesized that CLas and other bacterial pathogens would be resistant to the antibacterial activities of the PR proteins. Under specific circumstances, SAR inducers themselves might have direct antibacterial effects against CLas. For instance, SA exhibits antibacterial action in vitro against *Rhizobium meliloti* and *Agrobacterium tumefaciens* at significantly lower concentrations (5–25 M = 0.69–3.45 g/ml) [67,68]. CLas is a member of the Rhizobiaceae family and a close relative of Agrobacterium and Rhizobium [69]. The use of SAR inducers and the ensuing buildup of SA in a citrus tree could potentially have an inhibitory effect on CLas. A functioning SA hydroxylase that is encoded by CLas breaks down SA, reducing its effectiveness.

Application of SA and PAA not only induced the SA-dependent pathway but also the Jasmonic acid (JA) dependent pathway and also Ethylene. SA and PAA were able to induce the expression of 12-oxophytodienoate reductase 3 (*Opr_3*), allene oxide synthase (*Aos),* and Lipoxygenases (*Lox*) which is responsible for the synthesis of Jasmonic acid in JA pathways [45]. Another study showed that a stronger SA pathway was induced in the resistant cultivar Jindan (*Fortunella crassifolia* Swingle), while a stronger JA pathway was induced in the susceptible cultivar Wanjincheng orange. Not only SA and JA, the activity of defense enzymes also induced through the Ethylene pathway. Application of SA and PAA showed increasing expression of ethylene insensitive-2 (*Ein_*2), 1-aminocyclopropane-1-carboxylate synthase-1 (*Acs_1)*, and ethylene response-1 (*Ets_1*). Ethylene is produced from methionine by ACC synthase (*Acs*) and ACC oxidase (*Aco*) from its precursor 1-aminocyclopropane-1-carboxylic acid (ACC). The results indicate that SA and PAA may also increase the expression of *Acs*. Moreover, ethylene-responsive transcription factors will bind to the GCC-box pathogenesis-related promoter which is responsible for pathogens and increasing the expression of PR genes [[70], [71], [72]].

In conclusion, the control application of HLB disease in early-stage citrus plants is compulsory. Effective control methods could be obtained by the application of environmentally safe inducers such as SA and PAA. Even though this research was done in a greenhouse and without the use of actual field soil, it nonetheless represents a significant first step in understanding how grafted citrus trees react to CLas under carefully monitored settings. Additional research on field-grown grafted trees and metabolite analysis should both be included in future studies. Because it is challenging to compare infected and non-infected trees in an HLB-endemic environment, greenhouse research will continue to be a key for understanding the subtleties of CLas impacts.

## Funding

This Work was funded by the National Plan for Science, Technology, and Innovation (MAARIFAH), King Abdul-Aziz City for Science and Technology, Kingdom of Saudi Arabia, grant Number (14-BIO-627-02)**.**

## Author contribution statement

Arya Widyawan and Yasser E. Ibrahim: Conceived and designed the experiments, Performed the experiments, Analyzed and interpreted the data, Wrote the paper. Mohammed A. Al-Saleh; Mahmoud H. El Komy and Hathal M. Al Dhafer: Contributed reagents, materials, analysis tools or data, Wrote the paper.

## Data availability statement

Data included in article/supp. Material/referenced in article.

## Declaration of competing interest

The authors declare that they have no known competing financial interests or personal relationships that could have appeared to influence the work reported in this paper.

## References

[1] Andrade M., Li J., Wang N. (2020). *Candidatus* Liberibacter asiaticus: virulence traits and control strategies. Trop Plant Pathol.

[2] Da Graça J.V. (1991). Citrus greening disease. Annu. Rev. Phytopathol..

[3] Garnier M., Bové J.M. (1993). Citrus greening disease and the greening bacterium. Proceedings of 12th Conference IOCV, IOCV, Riverside.

[4] Huanglongbing Bové J.M. (2006). A destructive, newly-emerging, century-old disease of citrus. J. Plant Pathol..

[5] Teixeira D.C., Saillard C., Couture C., Martins E.C., Wulff N.A., Jagoueix S.E., Yamamoto P.T., Ayres A.J., Bove J.M. (2008). Distribution and quantification of *Candidatus* Liberibacter americanus, agent of huanglongbing disease of citrus in Sao Paulo State, Brasil, in leaves of an affected sweet orange tree as determined by PCR. Mol. Cell. Probes.

[6] Jagoueix S., Bove J.M., Garnier M. (1994). The phloem-limited bacterium of greening disease of citrus is a member of the subdivision of the proteobacteria. Int.J. Sys. Bacteriol..

[7] Hung T.H., Hung S.C., Chen C.N., Hsu M.H., Su H.J. (2004). Detection by PCR of *Candidatus* Liberibacter asiaticus, the bacterium causing citrus greening in vector psyllids: application to the study of vector pathogen relationship. Plant Pathol..

[8] Coletta-Filho H.D., Targon M.L.P.N., Takita M.A., De Negri J.D., Pompeu J., Machado M.A., do Amaral A.M., Muller G.W. (2004). First report of the causal agent of huanglongbing (‘*Candidatus* Liberibacter asiaticus’) in Brazil. Plant Dis..

[9] Batool A., Iftikhar Y., Mughal S.M. (2007). Citrus greening disease-a major cause of citrus decline in the world-a review. Hortic. Sci. (Stuttg.).

[10] Gottwald T.R., da Graça J.V., Bassanezi R.B. (2007). Citrus Huanglongbing: the pathogen and its impact. Plant Health Prog..

[11] Wang N., Trivedi P. (2013). Citrus huanglongbing: a newly relevant disease presents unprecedented challenges. Phytopathology.

[12] Wang N., Stelinski L.L., Pelz-Stelinski K.S., Graham J.H., Zhang Y. (2017). Tale of the Huanglongbing Disease Pyramid in the Context of the Citrus Microbiome Phytopathology.

[13] Alvarez S., Rohrig E., Solís D., Thomas M.H. (2016). Citrus greening disease (huanglongbing) in Florida: economic impact, management and the potential for biological control. Agric. Res..

[14] Boina D.R., Bloomquist J.R. (2015). Chemical control of the Asian citrus psyllid and of huanglongbing disease in citrus. Pest Manag. Sci..

[15] Grafton-Cardwell E.E., Stelinski L.L., Stansly P.A. (2013). Biology and management of asian citrus psyllid, vector of the huanglongbing pathogens. Annu. Rev. Entomol..

[16] Rouse R.E., Ozores-Hampton1 M., Roka F.M., Roberts P. (2017). Rehabilitation of huanglongbing affected citrus trees using severe pruning and enhanced foliar nutritional treatments. Hortscience.

[17] Stansly P.A., Arevalo H.A., Qureshi J.A., Jones M.M., Hendricks K., Roberts P.D., Roka F.M. (2014). Vector control and foliar nutrition to maintain economic sustainability of bearing citrus in Florida groves affected by huanglongbing. Pest Manag. Sci..

[18] Tansey J.A., Vanaclocha P., Monzo C., Jones M., Stansly P.A. (2017). Costs and benefits of insecticide and foliar nutrient applications to huanglongbing-infected citrus trees. Pest Manag. Sci..

[19] Zhao H.R., Sun U., Albrecht C., Padmanabhan A., Wang M.D., Coffey T., Girke Z., Wang T., Close J., Roose M., Yokomi R.K., Folimonova S., Vidalakis G., Rouse R., Bowman K.D., Jin H. (2013). Small RNA profiling reveals phosphorus deficiency as a contributing factor in symptom expression for citrus huanglongbing disease. Mol. Plant.

[20] Hu J., Wang N. (2016). Evaluation of the spatiotemporal dynamics of oxytetracycline and its control effect against citrus Huanglongbing via trunk injection. Phytopathology.

[21] Hu J., Jiang J., Wang N. (2018). Control of Citrus Huanglongbing via trunk injection of plant defense activators and antibiotics. Phytopathology.

[22] Yang C., Powell C.A., Duan Y., Shatters R., Zhang M. (2015). Antimicrobial nanoemulsion formulation with improved penetration of foliar spray through citrus leaf cuticles to control citrus huanglongbing. PLoS One.

[23] Xu M., Liang M., Chen J. (2013). Preliminary research on soil conditioner mediated citrus Huanglongbing mitigation in the field in Guangdong, China. Eur. J. Plant Pathol..

[24] Li J., Trivedi P., Wang N. (2016). Field evaluation of plant defense inducers for the control of citrus huanglongbing. Phytopathology.

[25] Graham J., Gottwald T., Setamou M. (2020). Status of huanglongbing (HLB) outbreaks in Florida, California and Texas. Trop. plant pathol..

[26] Stover E., Inch S., Richardson M.L., Hall D.G. (2016). Conventional citrus of some scion/rootstock combinations show field tolerance under high huanglongbing disease pressure. Hortscience.

[27] Al-Jumaili A., Ehsani R. (2015).

[28] Ehsani R., Dewdney M., Johnson E. (2016). Controlling HLB with thermotherapy: what have we learned so far?. Citrus Ind..

[29] Francis M.I., Redondo A., Burns J.K., Graham J.H. (2009). Soil application of imidacloprid and related SAR-inducing compounds produces effective and persistent control of citrus canker. Eur. J. Plant Pathol..

[30] Graham J.H., Leite R.P. (2004). Lack of control of citrus canker by induced systemic resistance compounds. Plant Dis..

[31] Graham J.H., Myers M.E. (2011). Soil application of SAR inducers imidacloprid, thiamethoxam, and acibenzolar-S-methyl for citrus canker control in young grapefruit trees. Plant Dis..

[32] Ananthakrishnan G., Choudhary N., Roy A., Sengoda V.G., Postnikova E., Hartung J.S., Stone A.L., Damsteegt V.D., Schneider W.L., Munyaneza J.E., Brlansky R.H. (2013). Development of primers and probes for genus and species-specific detection of ‘*Candidatus* Liberibacter species’ by real-time PCR. Plant Dis..

[33] Gottwald T.R., da Graça J.V., Bassanezi R.B. (2007). Citrus Huanglongbing: the pathogen and its impact. Plant Health Prog..

[34] Simko I., Piepho H.P. (2012). The area under the disease progress stairs: calculation, advantage, and application. Phytopathology.

[35] Doyle J.J., Doyle J.L. (1990). Isolation of plant DNA from fresh tissue. Focus.

[36] Kumar N., Ebel R.C., Roberts P.D. (2011). Antioxidant metabolism of grapefruit infected with *Xanthomonas axonopodis* pv. *citri*. Environ. Exp. Bot..

[37] Kavitha R., Umesha S. (2008). Regulation of defense-related enzymes associated with bacterial spot resistance in tomato. Phytoparasitica.

[38] Velikova V., Yordanov I., Edreva (2000). Oxidative stress and some antioxidant systems in acid rain treated bean plants: protective roles of exogenous polyamines. Plant Sci..

[39] Ramanathan A.P., Vidhyasekaran, Samiyappan R. (2001). Two pathogenesis related peroxidase in greengram (*Vigna radiate*(L) *wilczek*) leaves and cultured cells, induced by *Macrophomina phaseolina* (Tassi) gold and its elicitor. Microbial. Res..

[40] Groppa M.D.M.L., Tomaro, Fernandez M.E. (1999). Activity and expression of peroxidase from sunflower: effect of development. Rev. Bras. Fisiol. Vegetal.

[41] Kocacaliskan I., Demir Y., Kabar K. (1995). A study on polyphenol oxidase activity during seed germination. Phyton.

[42] Houssein A.A., Sabra F.S. (2005). Safening corn seedling in sandy soil from certain herbicides injuries using naphthalic anhydride and its effect on oxidative enzyme. J. Pest Cont. Environ. Sci..

[43] Livak K.J., Schmittgen T.D. (2001). Analysis of relative gene expression data using real-time quantitative PCR and the 2− ΔΔCT method. Methods.

[44] Dutt M., Barthe G., Irey M., Grosser J. (2015). Transgenic citrus expressing an Arabidopsis NPR1 gene exhibit enhanced resistance against Huanglongbing (HLB; Citrus Greening). PLoS One.

[45] Long Q., Xie Y., He Y., Li Q., Zou X., Chen S. (2019). Abscisic acid promotes jasmonic acid accumulation and plays a key role in citrus canker development. Front. Plant Sci..

[46] Kashyap K., Banu S. (2019). Characterizing ethylene pathway genes during the development, ripening, and postharvest response in Citrus reticulata Blanco fruit pulp. Turk. J. Bot..

[47] Li X., Ruan H., Zhou C., Meng X., Chen W. (2021). Controlling citrus huanglongbing: green sustainable development route is the future. Front. Plant Sci..

[48] Wang Y., Liu J.H. (2012). Exogenous treatment with salicylic acid attenuates occurrence of citrus canker in susceptible navel orange (*Citrus sinensis* Osbeck). J. Plant Physiol..

[49] Akbar H.A., Iqbal Z., Raza W., Ghazanfar M.U., Ahmed S. (2018). Management of citrus green mould through the use of Allelochemicals and salicylic acid. Journal of Innovative Sciences.

[50] Silva T.R., Valdman E., Valdman B., Leite S.G. (2007). Salicylic acid degradation from aqueous solutions using *Pseudomonas fluorescens* HK44: parameters studies and application tools. Braz. J. Microbiol..

[51] Sleiman M., Stawinoga M., Wang S., de Sainte-Claire P., Goupil P., Richard C. (2017). Photochemical transformation of the plant activator Acibenzolar-S-methyl in solution. J. Photochem. Photobiol., A: Chem.

[52] Sáenz-Pérez C.A., Osorio-Hernández E., Estrada-Drouaillet B., Castro-Nava S., Delgado-Martínez R., López-Badillo C.M., Rodríguez-Herrera R. (2020 14). Rootstock influence on growth and mineral content of *Citrus limon* and *Citrus sinensis* cv. valencia inoculated with *Candidatus* liberibacter asiaticus. Agronomy.

[53] Wu J.J., Huang J.W., Deng W.L. (2020). Phenylacetic Acid and Methylphenyl Acetate from the biocontrol bacterium *Bacillus mycoides* BM02 suppress spore germination in *Fusarium oxysporum* f. sp. *lycopersici*. Front. Microbiol..

[54] Akram W., Anjum T., Ali B. (2016). Phenylacetic acid is ISR determinant produced by *Bacillus fortis* IAGS162, which involves extensive re-modulation in metabolomics of tomato to protect against Fusarium wilt. Front. Plant Sci..

[55] Sumayo M.S., Son J.S., Ghim S.Y. (2018). Exogenous application of phenylacetic acid promotes root hair growth and induces the systemic resistance of tobacco against bacterial soft-rot pathogen Pectobacterium carotovorum subsp. carotovorum. Funct. Plant Biol..

[56] Zhang M., Duan Y., Zhou L., Turechek W.W., Stover E. (2010). Screening molecules for control of citrus huanglongbing using an optimized regeneration system for '*Candidatus* Liberibacter asiaticus'-infected periwinkle (*Catharanthus roseus*) cuttings. Phytopathology.

[57] Zhang M., Powell C.A., Zhou L., He Z., Stover E. (2011). Chemical compounds effective against the citrus Huanglongbing bacterium '*Candidatus* Liberibacter asiaticus' in planta. Phytopathology.

[58] Yang C., Powell C.A., Duan Y., Shatters R., Zhang M. (2015). Antimicrobial nanoemulsion formulation with improved penetration of foliar spray through citrus leaf cuticles to control citrus huanglongbing. PLoS One.

[59] Zhang M., Guo Y., Powell C.A., Doud M.S., Yang C. (2014). Effective antibiotics against ‘*Candidatus* liberibacter asiaticus’ in HLB-affected citrus plants identified via the graft-based evaluation. PLoS One.

[60] Zhang M.Q., Powell C.A., Benyon L.S., Zhou H., Duan Y.P. (2013). Deciphering the bacterial microbiome of citrus plants in response to '*Candidatus* liberibacter asiaticus'-infection and antibiotic treatments. PLoS One.

[61] Chopra I., Roberts M. (2001). Tetracycline antibiotics: mode of action, applications, molecular biology, and epidemiology of bacterial resistance. Microbiol. Mol. Biol. Rev..

[62] Zimmermann P., Curtis N. (2018). The effect of aspirin on antibiotic susceptibility. Expert Opin. Ther. Targets.

[63] Cao H., Bowling S.A., Gordon S., Dong X. (1994). Characterization of an Arabidopsis mutant that is nonresponsive to inducers of systemic acquired resistance. Plant Cell.

[64] Delaney T.P., Friedrich L., Ryals J.A. (1995). Arabidopsis signal transduction mutant defective in chemically and biologically induced disease resistance. Proc. Natl. Acad. Sci. U.S.A..

[65] Shah J., Tsui F., Klessig D.F. (1997). Characterization of a salicylic acid-insensitive mutant (sai1) of *Arabidopsis thaliana* identified in a selective screen utilizing the SA-inducible expression of the tms2 gene. Mol. Plant Microbe Interact..

[66] Ali S., Ganai B.A., Kamili A.N., Bhat A.A., Mir Z.A., Bhat J.A., Tyagi A., Islam S.T., Mushtaq M., Yadav P., Rawat S., Grover A. (2018). Pathogenesis-related proteins and peptides as promising tools for engineering plants with multiple stress tolerance. Microbiol. Res..

[67] Yuan Z.C., Edlind M.P., Liu P., Saenkham P., Banta L.M., Wise A.A., Ronzone E., Binns A.N., Kerr K., Nester E.W. (2008). The plant signal salicylic acid shuts down expression of the vir regulon and activates quormone-quenching genes in *Agrobacterium*. Proc. Natl. Acad. Sci. U.S.A..

[68] Anand A., Uppalapati S.R., Ryu C.M., Allen S.N., Kang L., Tang Y., Mysore K.S. (2008). Salicylic acid and systemic acquired resistance play a role in attenuating crown gall disease caused by Agrobacterium tumefaciens. Plant Physiol..

[69] Duan Y., Zhou L., Hall D.G., Li W., Doddapaneni H., Lin H., Liu L., Vahling C.M., Gabriel D.W., Williams K.P., Dickerman A., Sun Y., Gottwald T. (2009). Complete genome sequence of citrus huanglongbing bacterium, ‘Candidatus Liberibacter asiaticus’ obtained through metagenomics. Mol. Plant Microbe Interact..

[70] Zhou J., Tang X., Martin G.B. (1997). The Pto kinase conferring resistance to tomato bacterial speck disease interacts with proteins that bind a cis-element of pathogenesis-related genes. EMBO J..

[71] Fujimoto S.Y., Ohta M., Usui A., Shinshi H., Ohme-Takagi M. (2000). Arabidopsis ethylene-responsive element binding factors act as transcriptional activators or repressors of GCC box-mediated gene expression. Plant Cell.

[72] Sakuma Y., Liu Q., Dubouzet J.G., Abe H., Shinozaki K., Yamaguchi-Shinozaki K. (2002). DNA-binding specificity of the ERF/AP2 domain of Arabidopsis DREBs, transcription factors involved in dehydrationand cold-inducible gene expression. Biochem. Biophys. Res. Commun..

