# Supplementary material for: Potential of resistance inducers for citrus huanglongbing management via soil application and assessment of induction of pathogenesis-related protein genes
Source: Heliyon. 2023 Sep 1;9(9):e19715. doi: 10.1016/j.heliyon.2023.e19715 (PMC10558989; doi:10.1016/j.heliyon.2023.e19715)
Supplement: Supplementary data [file mmc1.docx]

Supplementray Figure (1) A graphical figure showing the experimental design

|  | 1Ai | 2Ai | 1Ci | 2Bi | 1Bi | 2Ci |
| --- | --- | --- | --- | --- | --- | --- |
|  | 2Avi | 1Avi | 2Cvi | 1Cvi | 2Bvi | 1Bvi |
|  | 1Aiii | 2Aiii | 1Ciii | 2Biii | 1Biii | 2Ciii |
|  | 2Aii | 1Aii | 2Civ | 1Civ | 2Biv | 1Biv |
|  | 1Aiv | 2Aiv | 1Civ | 2Biv | 1Biv | 2Civ |
| Blok I | 2Aiv | 1Aiii | 2Ciii | 1Ciii | 2Biii | 1Biii |
|  | 1Av | 2Av | 1Cv | 2Bv | 1Bv | 2Cv |
|  | 2Aiii | 1Ai | 2Cii | 1Cii | 2Bii | 1Bii |
|  | 1Avi | 2Avi | 1Cvi | 2Bvi | 1Bvi | 2Cvi |
|  | 2Ai | 1Av | 2Ci | 1Ci | 2Bi | 1Bi |
|  | 1Aii | 2Aii | 1Cii | 2Bii | 1Bii | 2Cii |
|  | 2Av | 1Aiv | 2Cv | 1Cv | 2Bv | 1Bv |
|  |  |  |  |  |  |  |
|  | 1Ai | 2Ai | 1Ci | 2Bi | 1Bi | 2Ci |
|  | 2Avi | 1Avi | 2Cvi | 1Cvi | 2Bvi | 1Bvi |
|  | 1Aiii | 2Aiii | 1Ciii | 2Biii | 1Biii | 2Ciii |
|  | 2Aii | 1Aii | 2Civ | 1Civ | 2Biv | 1Biv |
|  | 1Aiv | 2Aiv | 1Civ | 2Biv | 1Biv | 2Civ |
| Block II | 2Aiv | 1Aiii | 2Ciii | 1Ciii | 2Biii | 1Biii |
|  | 1Av | 2Av | 1Cv | 2Bv | 1Bv | 2Cv |
|  | 2Aiii | 1Ai | 2Cii | 1Cii | 2Bii | 1Bii |
|  | 1Avi | 2Avi | 1Cvi | 2Bvi | 1Bvi | 2Cvi |
|  | 2Ai | 1Av | 2Ci | 1Ci | 2Bi | 1Bi |
|  | 1Aii | 2Aii | 1Cii | 2Bii | 1Bii | 2Cii |
|  | 2Av | 1Aiv | 2Cv | 1Cv | 2Bv | 1Bv |
|  |  |  |  |  |  |  |
|  | 1Ai | 2Ai | 1Ci | 2Bi | 1Bi | 2Ci |
|  | 2Avi | 1Avi | 2Cvi | 1Cvi | 2Bvi | 1Bvi |
|  | 1Aiii | 2Aiii | 1Ciii | 2Biii | 1Biii | 2Ciii |
|  | 2Aii | 1Aii | 2Civ | 1Civ | 2Biv | 1Biv |
|  | 1Aiv | 2Aiv | 1Civ | 2Biv | 1Biv | 2Civ |
| Block III | 2Aiv | 1Aiii | 2Ciii | 1Ciii | 2Biii | 1Biii |
|  | 1Av | 2Av | 1Cv | 2Bv | 1Bv | 2Cv |
|  | 2Aiii | 1Ai | 2Cii | 1Cii | 2Bii | 1Bii |
|  | 1Avi | 2Avi | 1Cvi | 2Bvi | 1Bvi | 2Cvi |
|  | 2Ai | 1Av | 2Ci | 1Ci | 2Bi | 1Bi |
|  | 1Aii | 2Aii | 1Cii | 2Bii | 1Bii | 2Cii |
|  | 2Av | 1Aiv | 2Cv | 1Cv | 2Bv | 1Bv |

Note :

| Code | Description |
| --- | --- |
| 1 | *C. aurantium* |
| 2 | *C. volkameriana* |
| A | Mandarin |
| B | Sweet Orange |
| C | Mexican lime |
| i | Salicylic acid |
| ii | Phenyl Aceticacid |
| iii | Oxytetracycline |
| iv | Salicylic acid + Oxytetracycline |
| v | Phenyl Aceticacid + Oxytetratcycline |
| vi | Water |

**Supplementary Table (1). Cq value of *Candidatus* liberibacter asiacticus in different citrus seedlings prior to treatments**

|  | Cultivars | Mandarin | | Sweet Orange | | Mexican lime | |
| --- | --- | --- | --- | --- | --- | --- | --- |
|  | Rootstock/replicate no. | *C. aurantium* | *C. volkameriana* | *C. aurantium* | *C. volkameriana* | *C. aurantium* | *C. volkameriana* |
|  | Tree 1* | 30.25 | 35.12 | 33.00 | 30.77 | 31.91 | 30.22 |
|  | Tree 2 | 30.27 | 35.00 | 32.35 | 30.69 | 31.53 | 30.50 |
|  | Tree 3 | 31.99 | 35.17 | 31.16 | 25.47 | 32.00 | 33.89 |
|  | Tree 4 | 30.50 | 35.00 | 31.12 | 25.84 | 31.35 | 33.32 |
|  | Tree 5 | 31.12 | 33.36 | 33.32 | 29.00 | 30.33 | 33.05 |
|  | Tree 6 | 30.12 | 33.57 | 32.33 | 29.38 | 31.86 | 33.00 |
|  | Tree 7 | 31.80 | 33.43 | 35.02 | 31.10 | 32.23 | 31.71 |
|  | Tree 8 | 29.30 | 33.00 | 34.35 | 31.33 | 32.45 | 32.00 |
|  | Tree 9 | 30.42 | 33.99 | 35.20 | 26.29 | 32.00 | 33.16 |
|  | Tree 10 | 29.43 | 32.69 | 34.44 | 26.13 | 31.30 | 33.00 |
|  | Tree 11 | 35.12 | 33.00 | 31.91 | 25.25 | 31.77 | 30.25 |
|  | Tree 12 | 35.00 | 33.35 | 31.53 | 25.27 | 32.69 | 31.27 |
|  | Tree 13 | 31.74 | 35.16 | 34.00 | 31.99 | 32.47 | 31.99 |
|  | Tree 14 | 32.00 | 35.12 | 33.35 | 31.00 | 31.84 | 32.34 |
|  | Tree 15 | 33.36 | 32.32 | 30.33 | 22.12 | 31.00 | 33.12 |
|  | Tree 16 | 31.57 | 33.33 | 31.86 | 21.12 | 29.38 | 33.21 |
|  | Tree 17 | 33.43 | 33.02 | 33.23 | 31.80 | 31.10 | 31.80 |
|  | Tree 18 | 31.50 | 33.35 | 32.45 | 31.00 | 31.33 | 32.00 |
|  | Tree 19 | 30.99 | 33.27 | 31.00 | 32.42 | 31.29 | 30.42 |
|  | Tree 20 | 30.69 | 33.44 | 30.30 | 32.43 | 32.13 | 29.43 |
|  | Tree 21 | 31.91 | 31.91 | 35.12 | 28.91 | 30.22 | 31.91 |
|  | Tree 22 | 31.53 | 31.53 | 35.00 | 28.53 | 31.50 | 31.53 |
|  | Tree 23 | 32.00 | 34.00 | 31.74 | 32.00 | 33.89 | 31.00 |
|  | Tree 24 | 31.35 | 34.35 | 32.00 | 31.35 | 32.32 | 31.35 |
|  | Tree 25 | 30.33 | 30.33 | 33.36 | 19.33 | 33.05 | 30.33 |
|  | Tree 26 | 31.86 | 31.86 | 32.57 | 19.86 | 33.00 | 31.86 |
|  | Tree 27 | 32.23 | 32.23 | 33.43 | 24.23 | 32.71 | 35.03 |
|  | Tree 28 | 32.45 | 32.45 | 33.00 | 24.45 | 32.00 | 34.45 |
|  | Tree 29 | 32.33 | 34.00 | 30.99 | 23.86 | 35.23 | 34.00 |
|  | Tree 30 | 30.30 | 33.30 | 30.69 | 24.30 | 35.00 | 30.30 |
|  | Tree 31 | 30.00 | 32.91 | 30.25 | 25.77 | 30.22 | 30.25 |
|  | Tree 32 | 30.35 | 33.53 | 29.27 | 25.69 | 31.50 | 29.27 |
|  | Tree 33 | 31.16 | 34.00 | 31.99 | 23.47 | 33.89 | 31.99 |
|  | Tree 34 | 31.12 | 34.35 | 32.12 | 23.84 | 31.32 | 32.00 |
|  | Tree 35 | 21.32 | 33.33 | 35.12 | 23.00 | 35.05 | 31.12 |
|  | Tree 36 | 20.33 | 32.86 | 34.12 | 23.38 | 35.00 | 30.12 |

***36 seedlings (Tree 1-36) for each cultivar per rootstock were tested against CLas**

| **Supplementary Table 2. Percent (%) changes of Cq value of Candidatus liberibacter asiacticus in different citrus seedling after treatments with Salicylic acid (SA) and Phenyl Acetic acid (PAA) with or without Oxytetracycline (OTC).** |
| --- |

| Treatments | Mandarin | | Sweet Orange | | Mexican lime | |
| --- | --- | --- | --- | --- | --- | --- |
|  | *C. aurantium* | *C. volkameriana* | *C. aurantium* | *C. volkameriana* | *C. aurantium* | *C. volkameriana* |
| SA – Seedling 1 | 28.91 | 11.06 | 22.42 | 18.18 | 13.69 | 22.77 |
| SA – Seedling 2 | 27.80 | 11.56 | 23.00 | 18.36 | 13.00 | 21.00 |
| SA – Seedling 3 | 25.33 | 12.00 | 20.00 | 24.00 | 8.50 | 22.56 |
| SA – Seedling 4 | 25.00 | 12.25 | 19.50 | 24.06 | 9.00 | 21.23 |
| SA – Seedling 5 | 22.00 | 13.80 | 17.50 | 28.00 | 2.00 | 19.34 |
| SA – Seedling 6 | 21.75 | 13.44 | 17.46 | 28.50 | 1.89 | 19.30 |
| PAA – Seedling 7 | 21.92 | 9.21 | 15.09 | 10.93 | 14.71 | 21.60 |
| PAA – Seedling 8 | 20.52 | 10.05 | 14.70 | 11.50 | 14.00 | 21.34 |
| PAA – Seedling 9 | 18.20 | 11.34 | 19.81 | 18.05 | 8.54 | 22.13 |
| PAA – Seedling 10 | 18.72 | 10.32 | 19.23 | 19.00 | 7.09 | 23.00 |
| PAA – Seedling 11 | 14.00 | 12.21 | 23.32 | 25.05 | 3.78 | 22.05 |
| PAA – Seedling 12 | 14.71 | 11.43 | 24.52 | 25.31 | 0.68 | 22.51 |
| OTC – Seedling 13 | 11.04 | 16.90 | 18.00 | 65.08 | 21.13 | 14.71 |
| OTC – Seedling 14 | 12.00 | 17.00 | 18.67 | 65.67 | 21.56 | 15.00 |
| OTC – Seedling 15 | 45.00 | 20.25 | 21.00 | 78.42 | 18.56 | 23.75 |
| OTC – Seedling 16 | 47.85 | 21.00 | 21.15 | 77.78 | 18.88 | 24.00 |
| OTC – Seedling 17 | 84.05 | 23.05 | 24.00 | 90.87 | 16.07 | 32.55 |
| OTC – Seedling 18 | 84.66 | 23.55 | 24.28 | 91.76 | 16.37 | 32.76 |
| SA + OTC – Seedling 19 | 22.66 | 1.27 | 13.31 | 0.34 | 10.70 | 25.42 |
| SA + OTC – Seedling 20 | 22.23 | 2.50 | 13.00 | -0.90 | 10.23 | 25.22 |
| SA + OTC – Seedling 21 | 17.07 | 6.78 | 12.37 | -2.66 | 7.06 | 25.00 |
| SA + OTC – Seedling 22 | 18.00 | 7.05 | 12.87 | -2.87 | 7.21 | 24.30 |
| SA + OTC – Seedling 23 | 11.07 | 12.05 | 11.22 | -4.67 | 4.00 | 24.07 |
| SA + OTC – Seedling 24 | 11.43 | 11.43 | 11.43 | -5.68 | 3.38 | 24.50 |
| PAA + OTC – Seedling 25 | 28.19 | 10.32 | 17.60 | -7.34 | 6.65 | 24.66 |
| PAA + OTC – Seedling 26 | 29.05 | 11.05 | 16.87 | -8.05 | 5.78 | 24.07 |
| PAA + OTC – Seedling 27 | 31.07 | 18.50 | 14.26 | -9.25 | -2.78 | 23.08 |
| PAA + OTC – Seedling 28 | 30.34 | 18.70 | 14.51 | -9.78 | -3.05 | 22.87 |
| PAA + OTC – Seedling 29 | 32.12 | 26.80 | 11.05 | -10.32 | -12.78 | 21.97 |
| PAA + OTC – Seedling 30 | 32.50 | 27.08 | 11.43 | -11.15 | -13.25 | 21.37 |
| Water – Seedling 31 | -7.02 | -7.20 | -6.15 | -5.80 | -8.25 | -3.83 |
| Water – Seedling 32 | -6.98 | -7.05 | -5.98 | -5.12 | -7.89 | -4.23 |
| Water – Seedling 33 | -5.25 | -6.00 | -6.89 | -4.12 | -6.50 | -4.96 |
| Water – Seedling 34 | -5.56 | -6.02 | -7.08 | -4.50 | -6.77 | -5.05 |
| Water – Seedling 35 | -3.87 | -6.08 | -8.06 | -2.98 | -5.00 | -5.88 |
| Water – Seedling 36 | -4.09 | -6.77 | -8.25 | -3.20 | -5.30 | -6.25 |
